# Supplementary material for: Insights into Predictors of Vaccine Hesitancy and Promoting Factors in Childhood Immunization Programs—A Cross-Sectional Survey in Cameroon
Source: Int J Environ Res Public Health. 2022 Feb 26;19(5):2721. doi: 10.3390/ijerph19052721 (PMC8910338; doi:10.3390/ijerph19052721)
Supplement: Supplementary file 1 [file ijerph-19-02721-s001.zip › ijerph-1527713-supplementary.pdf]

International Journal of
Environmental Research and Public Health

MDPI

1.1. Validation of the Logistic Regression Model

A logistic regression model was used to find out important predictor(s) of complete vaccination failure from a set of identified variables. Initially, the following hypotheses were checked by means of the Chi-square test:

- null hypothesis H_0: $\beta_i = 0$ for all i, i=1; variables do not predict maternal decision about childhood vaccination status.
- alternative hypothesis H_A: β_i = coefficient for $x_i \neq 0$ for at least 1 coefficient,

  x_i = variable; variables predict maternal decision about childhood vaccination status.

The value of the Chi-square test model is the difference between the null model (model-intercept) and the full model (model with variables). The results from the Chi-square test include a p-value that measures how compatible the data are with the null hypothesis. A high p-value suggests that data are likely to be true with the null hypothesis. A low p-value suggests that the sample presents enough evidence to reject the null hypothesis for the studied population. In case the null hypothesis is true, variables have no influence on maternal decision about childhood vaccination status. Otherwise the null hypothesis is rejected in favor of the alternative hypothesis and the analysis can continue in order to find the model that will better fit the data. Different steps have been used to assess which model fits the data better and does a better job at predicting decisions.

Null-model (model-intercept)

The first step is called step 0. The step 0 (Table 1)-(Table 3) expresses the statistics of the null-model (model-intercept) i.e. the model without considering variables.

Table S1. Classification Table [a, b] – Model-Intercept.

Observed			Predicted		
			Completely vaccinated		Percentage Correct
			0	1	
Step 0	Completely vaccinated	**0**	0	64	0.0
		1	0	96	100.0
	Overall Percentage				60.0

[a] Constant is included in the model
[b] The cut value is 0.5

Table S2. Variables in the Equation – Model-Intercept.

		Regression Coefficient B	S.E.	Wald	df (degree of freedom)	Sig.	Exp(B)
**Step 0**	Constant	0.405	0.161	6.313	1	0.012	1.500

Under variables in the Equation (Table 2), the intercept-only model is ln (odds) =0.405. If both sides of the expression are exponentiated, the predicted odds of deciding for complete vaccination are 1.5. Since 96 of the mothers chose complete childhood vaccination and 64 decided to refuse complete vaccination, the observed odds are 96/64=1.5. To check if in this study childhood vaccination status depends on sociodemographic factors, the Chi-square test was performed. As a result, the regression coefficient was tested with Wald chi square test to see if it is equal to zero. Since p-value is 0.012, the coefficient is significantly different from zero (Table 2).

According to the results of the overall Chi-square test (Table 4), the null hypothesis is rejected since p-value < 0.0001. Therefore, the analysis was continued.

Table S3. Variables not in the Equation -Model-Intercept.

			Score	df	Sig.
**Step 0**	**Variables**	single	4.422	1	0.035
		Muslim	10.435	1	0.001
		Poor Knowledge of VPDs	4.875	1	0.027
		Good Knowledge of VPDs	6.254	1	0.001
		Start at birth	5.228	1	0.022
		Mastering Schedule	8.178	1	0.004
	Overall Statistics		25.964	5	0.0001

Table S4. Omnibus Test of Model Coefficients – Overall Chi-square test.

		Chi-square	df	Sig.
**Step1**	Step	29.317	5	0.0001
	Block	29.317	5	0.0001
	Model	29.317	5	0.0001

Full model (model with variables)

Under model summary (Table 5), it is observed that when all variables are considered simultaneously (full model), the -2 Log Likelihood statistic is 186.047. This statistic shows the performance of the model to predict decisions. The better model is the one with the smaller – 2 Log Likelihood. Since Chi-square is equal to 29.317, the – 2 Log Likelihood statistic for the model that has only the intercept is 215.364. The statistic has dropped to 186.047 indicating that the model with variables (full model) performs better than the model-intercept (null model).

Table S5. Model Summary – Full Model.

Step	-2 Log-Likelihood	Cox & Snell R-Square	Nagelkerkes R-Square
1	186.047 [a]	0.167	0.226

[a.] Estimation terminated at the iteration number 5 because parameter estimates changed by less than 0.001

1.1.1.1 Construction of the Full Models

The variables in the equation table (Table 6) contain the coefficients for the fitted line and other information about the coefficients. It shows that all the coefficients are different from zero. The Wald chi square statistic has also been given. It tests the unique influence of each variable in the context of the other variables: holding constant the other variables; removing any overlap between variables.

Each variable met the conventional 0.05 standard for statistical significance, except for the variables “single” and “mastering schedule”.

In this study, the Wald chi square test was considered to be too conservative; that means, lacking enough power. An alternative was to test the significance of each variable by entering it stepwise into the null model and testing the significance of the model fitting criteria for each expanded model.

The forward selection method was used to construct different models (step1, step 2, and step 3) (Table 7)-(Table 10). This also enables to assess which expanded model would be preferable.

Table S6. Variables in the Equation.

		Regression Coefficient B	S.E.	Wald	df	Sig.	Exp(B)	95% C.I. for EXP(B)	
								Lower	Upper
**Step 1** [a]	**Single**	**0.427**	**0.678**	**0.397**	**1**	**0.529**	1.533	0.406	5.788
	Muslim	-1.500	0.559	7.202	1	0.007	0.223	0.075	0.667
	Poor Knowledge of VPDs	-1.074	0.517	4.318	1	0.038	0.342	0.124	0.941
	Start at birth	1.550	0.511	9.210	1	0.002	4.713	1.732	12.829
	Good Knowledge of VPDs	1.623	0.502	8.352	1	0.001	5.324	1.851	11.524
	Mastering Schedule	0.591	0.534	1.227	1	0.268	1.806	0.635	5.138
	Constant	0.300	0.576	0.271	1	0.603	1.349		

[a.] variable(s) entered on step 1: Single. Muslim. Poor Knowledge. Start at birth. Mastering Schedule. Good Knowledge of VPDs

The variables were added one-at-a-time from the model until only variables with statistically significant p-values remained. The steps of the forward-selection are shown under the table "Information Criteria" (Table 10).

Table S7. Classification Table [a] – Stepwise Regression.

Observed			Predicted		
			Completely vaccinated		Percentage Correct
			0	1	
**Step 1**	**Completely vaccinated**	**0**	0	64	0
		1	0	96	100
	Overall Statistics				60
Step 2	Completely vaccinated	0	16	48	25
		1	0	96	100
	Overall Statistics				70
Step 3	Completely vaccinated	0	26	38	40.6
		1	5	91	94.8
	Overall Statistics				73.1

[a.] The cut value is 0.5

Table S8. Variable in the Equation – Stepwise Regression.

		Regression Coefficient B	S.E.	Wald	df	Sig.	EXP(B)	95% C.I. for EXP(B)	
								Lower	Upper
**Step1** [a]	**Muslim**	**-1.299**	**0.417**	**9.717**	**1**	**0.002**	0.273	0.120	0.617
	Constant	1.386	0.373	13.837	1	0.0001	4.000		
Step 2 [b]	Muslim	-1.769	0.485	13.275	1	0.0001	0.171	0.066	0.442
	Good Knowledge of VPDs	1.526	0.493	9.589	1	0.002	4.600	1.751	12.083
	Constant	0.524	0.453	1.338	1	0.247	1.689		
Step 3 [c]	Muslim	-1.850	0.496	13.906	1	0.0001	0.157	0.060	0.416
	Poor Knowledge of VPDs	-1.183	0.514	5.306	1	0.021	0.306	0.112	0.838
	Good Knowledge of VPDs	1.565	0.504	9.642	1	0.002	4.782	1.781	12.838
	Constant	0.726	0.472	2.364	1	0.124	2.068		

a. Variable(s) entered on step 1: Muslim

b. Variable(s) entered on step 2: Good Knowledge of VPDs

c. Variable(s) entered on step 3: Poor Knowledge of VPDs

1.1.1.2 Selection of the Best Model

The forward selection method resulted in a reduction in the expanded model (step 3) of the Akaike's Information Criterion (AIC), Bayesian Information Criterion (BIC) and -2 Log Likelihood statistic respectively from 85.71 to 64.16, from 88.8 to 76.5 and from 83.709 to 56.164 (Table 11). The best model is the model with the smallest AIC or BIC value and –2 Log Likelihood statistic.

Table S9. Model Summary – Stepwise Regression.

Step	-2 Log-Likelihood	Cox & Snell R-Square	Nagelkerkes R-Square
1	204.243[a]	0.067	0.091
2	193.402[a]	0.128	0.173
3	187.819[b]	0.158	0.214

a. Estimation terminated at iteration number 4, because parameter estimates changed by less than 0.001

b. Estimation terminated at iteration number 5, because parameter estimates changed by less than 0.001

Table S10. Step Summary - Information Criteria.

Model	Action	Effect(s)	Model Fitting Criteria			Effect Selection Tests		
			AIC	BIC	-2 Log-Likelihood	Chi-square [a]	degree of freedom	Significance
**0**	**Entered**	Constant	85.70	88.78	83.70	.		
1	Entered	Muslim	76.58	82.73	72.58	11.12	1	0.001
2	Entered	Good Knowledge of VPDs	67.74	76.97	61.74	10.84	1	0.001
3	Entered	Poor Knowledge of VPDs	64.16	76.46	56.14	5.58	1	0.018

Stepwise Method: Forward Selection

[a.] The Chi-Square for entry is based on the Likelihood-Ratio-Test

Table S11. Information for Model Fitting Criteria.

**Model**	**Model Fitting Criteria**			**Likelihood-Ratio-Tests**		
	**AIC**	**BIC**	**-2 Log-Likelihood**	**Chi-Square**	**degree of freedom**	**Significance**
Only Intercept	85.70	88.78	83.70			
Final	64.16	76.46	56.16	27.54	3	0.0001

Therefore the final model (step 3) shows the smallest value of the AIC, the BIC, the – 2 Log Likelihood statistics (Table 9)-(Table 11). Also, in the final model (step 3), the R-square statistics (error reduction) and the overall success rate have increased in comparison to the other models (step 0, step 1 and step 2).

Simultaneously the overall success rate in classification has improved from 60% in the intercept-model to 73.1% in the final model (step 3) (Table 7). Therefore, the final model (step 3) is better suited to explain the decision of mothers about childhood vaccination status.

1.1.1.3 Goodness-of-fit of the Selected Model

The evaluation of the fit of the logistic regression model employed in this study (model step 3) was done by generating a Receiver Operating Characteristic (ROC) curve by means of SPSS program. The ROC as a measure of goodness-of-fit depends on the simultaneous measure of sensitivity and specificity for all potential cutoff points. The area under the ROC curve (AUC) that indicates the discriminative ability of the prediction model was found to be 0.73 with 95% confidence interval (0.653, 0.818) (Figure 1)(Table 12). Hence, the AUC is significantly different from 0.5 since p-value is lower than 0.0001 indicating that the logistic regression by means of the final model (step 3) predicts maternal decision about childhood vaccination status significantly than by chance.

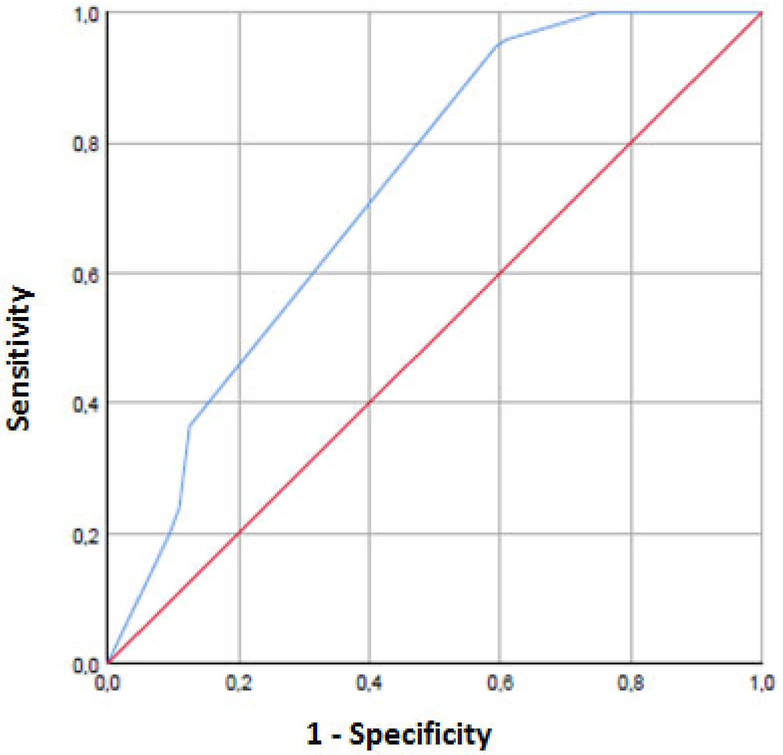

Figure 1. Receiver Operating Characteristic (ROC) curve – Final Model (step 3).

Table S12. Area Under the Curve – Test Result Variable(s) – Predicted probability.

**Area**	**Standard Error** [a]	Asymptotic Sig.[b]	Asymptotic 95% Confidence Interval	
			Lower Bound	Upper Bound
**0.728**	**0.042**	0.0001	0.645	0.810

a. Under the nonparametric assumption

b. Null hypothesis: true area = 0.5
